# Gridography tractography reveals communication between key areas from global workspace and integrated information theories of consciousness

**DOI:** 10.1038/s41598-025-31016-y

**Published:** 2025-12-30

**Authors:** Nicolas Lori, José Machado

**Affiliations:** 1 Algoritmi/LASI, University of Minho, Braga, 4710-057 Portugal; 2 Faculty of Science and Technology, University of Azores, Ponta Delgada, 9500-321 Portugal

**Keywords:** Tractography, Diffusion MRI, Brain, Integrated information theory, Global workspace theory, Consciousness, Imaging techniques, Data acquisition, Computational science

## Abstract

The study of consciousness is gaining importance in both neuroscience and the development of Artificial Intelligence (AI). We show here that an advanced White Matter (WM) tractography method, termed gridography, can explore the potential integration of two prominent theories of consciousness: Global Workspace Theory (GWT) and Integrated Information Theory (IIT). Using gridography on high-resolution diffusion MRI data from the Human Connectome Project, we demonstrate that gridography obtains WM connections between the anterior brain regions associated with GWT and posterior regions linked to IIT in a form which agrees with the Epiontic Consciousness Theory (ECT), which is an intermediary theory between GWT and IIT. We evaluate how experimental gridography data aligns with the physiological structures implicated in consciousness by analyzing: (i) Information characteristics of consciousness theories; (ii) Improvement of diffusion MRI tractography by use of gridography; (iii) Expected gridography results based on consciousness theory. Our findings suggest that these connections, particularly those of the Superior Longitudinal Fasciculus (SLF), support a ECT unified model of consciousness integrating aspects of both the primarily epistemic GWT and the primarily ontic IIT. This study proposes a novel framework that could reconcile existing theoretical divisions between GWT and IIT through the use of the ECT approach.

## Introduction

**Fig. 1:**
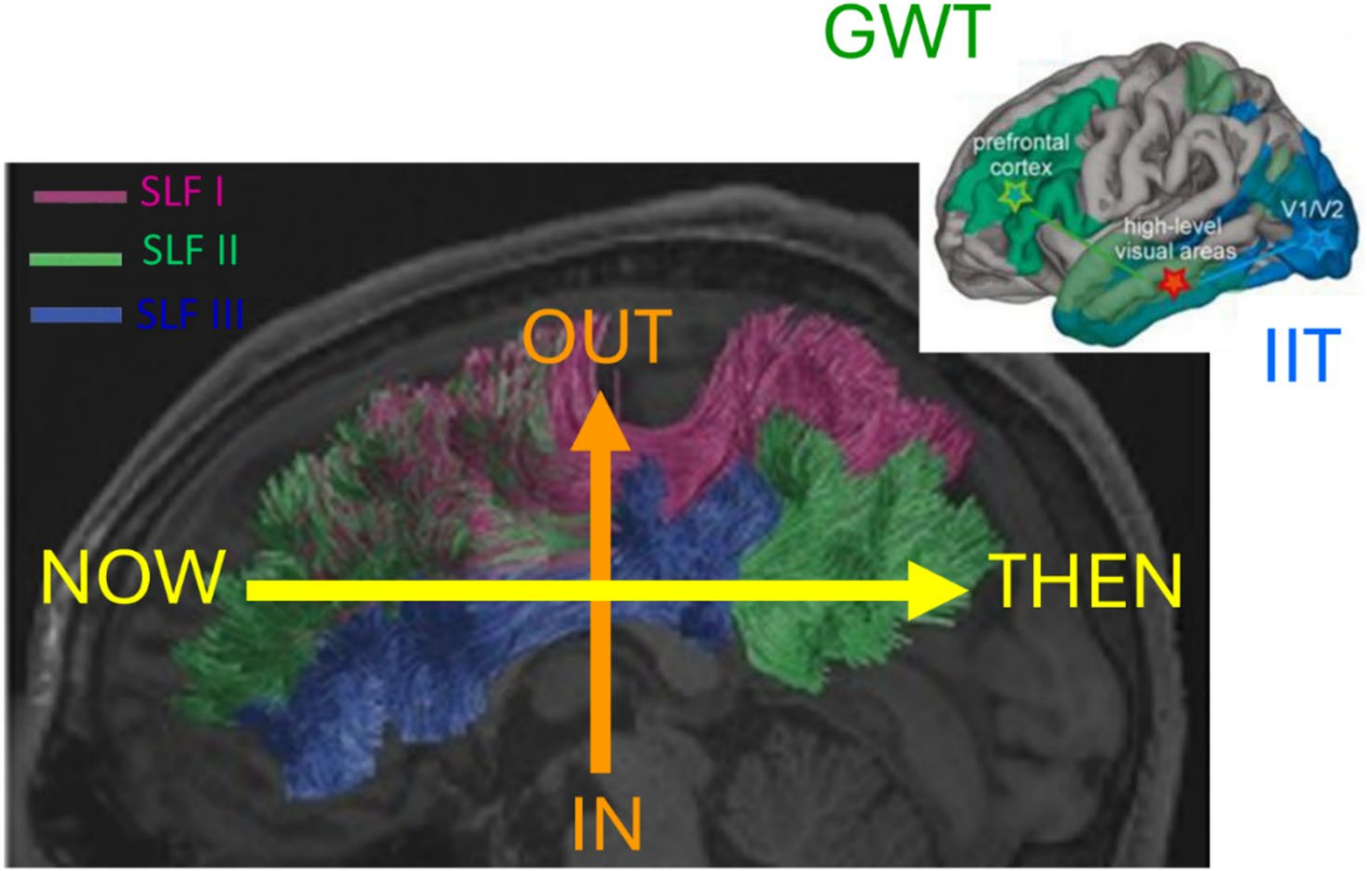
Integration between IIT and GWT requires a WM connection joining their respective brain regions obtained in ref^3^., and we can use ref^4^. combined with ref^26^. to propose that the Superior Longitudinal Fasciculus (SLF) is the WM that best suits the requirements. Inserted in upper right corner is the anatomical location of the consciousness-related areas for the GWT ToC (green) and IIT ToC (blue) based on ref^3^. Main figure is a simplified relation between brain 3-axes approach and brain function according to contemporary neuroscience^4^, overlaid on the location of the SLF, divided between its sections, as described in ref^26^. : SLF I, SLF II, SLF III.

The two main Theory of Consciousness (ToC) approaches are Global Workspace Theory (GWT)^1^ which locates consciousness mostly in the prefrontal cortex (see Fig. 1 upper right insert) and Integrated Information Theory (IIT)^2^ which locates consciousness mostly in the posterior aspects of the brain (see Fig. 1 upper right insert). The GWT and IIT ToC approaches were experimentally tested^3^ and some agreement with both ToC approaches have been validated, with IIT being the most validated, but it should be noticed that neither the sustained synchronization in posterior cortex expected by IIT approach nor the ignition at stimulus offset expected by GWT approach were observed. The Epiontic Consciousness Theory (ECT) approach^4^ was an attempt to describe the GWT and IIT approaches as simplifications of an integrated approach to the role of information in consciousness. The ECT integrated approach^4^ consists in integrating the mostly epistemic GWT to the mostly ontic IIT by use of the epiontic (meaning epistemic + ontic) concept which signifies that the ontic aspect can only survive by the broadcasting of its epistemic aspect and vice-versa.

The integration of ethical considerations in the design and development of Artificial Intelligence (AI) systems^5,6^ is crucial to ensure these technologies align with challenges such as bias, privacy, and manipulation^7,8^; and to maximize its implementation on society^9^ it is useful the assessment made here of how consciousness can arise in systems. The relevance of computer decision-making in society has increased steadily over the past decade^10^, which has elevated the importance of ethics in AI, particularly as human supervision in AI systems diminishes^11–13^ both for top-down approaches^14^ and bottom-up approaches^15^. Understanding consciousness in AI necessitates a deep exploration of the topology of neuronal connections’ role in consciousness, especially within the White Matter (WM), which comprises the major neuronal bundles connecting various brain regions. In humans, WM connections are typically assessed using tractography technologies.

The epiontic concept was first developed for an explanation of how single-possibility state objective reality is obtained from a multiple-possibilities state (in quantum mechanics called a “pure state”) by a Darwinian Evolution-based approach to quantum mechanics called Quantum Darwinism (QD)^16^, so the ECT approach can be conceived as being mathematically related to the Orchestrated Objective Reduction (OOR) approach to consciousness^17^ which uses quantum mechanics, but in the ECT approach the information behaves similarly to what occurs in quantum mechanics without requiring that neurons are in a quantum mechanics “pure state” as such requirement is likely to be impossible due to its temperature (although recent data questions that impossibility)^18^. Instead, the ECT approach assumes a wave-based information transportation + dissipation perspective that is mathematically similar to QD, but without requiring that neurons are in a quantum mechanics multi-possibilities state (a.k.a. “pure state”). In the ECT approach, it is assumed that neuronal states lose the coherence of their “multiple-possibilities of information-interconnection” state in a form that has mathematical similarity to the decoherence process of QD. In QD approach to quantum mechanics^16^, the successful transmission of information is the imprinting of the information about the system into the environment states, with such imprinting converting through a Darwinian process the multi-possibilities vector state into a single-possibility classically objective state^16^, and the ECT assumes a mathematically similar approach. In ECT, the survival of neuronal states occurs due to the flow of the information onto the cerebral environment. This study aims to examine the relationship between consciousness and WM tractography data, using an advanced method called gridography to potentially bridge gaps in our understanding of consciousness as modeled by both AI and biological systems.

## Theory

### Information characteristics of consciousness theories

Although the two main contemporary approaches to ToC^3^ are GWT and IIT, there are two other approaches that are also of great relevance^19^, specifically, Higher-Order Theories (HOT) and Predictive Processing Theories (PPT), with PPT often being bundled together with Reentry Theories (RT). The core claims for each of these ToC are, respectively^19^: (i) in HOT the mental state is conscious due to being the target of a certain type of meta-representation state, with a meta-representation being links between representations; (ii) in GWT the basic concept is like that of “blackboard” architectures in AI, wherein the conscious mental states are those that are globally available to a very large range of cognitive processes, with an emphasis on such access being obtained through ignition and broadcast; (iii) in IIT the starting point is the proposal of mathematical axioms about the phenomenological aspect of the conscious experience, with the physical systems where the properties derived from such axioms occur instantiating consciousness, with consciousness intensity being assessed by both a parameter Φ that mostly measures how much the whole has more cause-effect information than the parts and by the “shapes” of high-dimensional conceptual structures representing the mechanistic aspect of the cause-effect system; (iv) in PPT the distinction between conscious and unconscious states is a “best guess” about the causes of sensory signals during perceptual inference, being therefore more a general account of how the brain functions during consciousness processes rather than a ToC.

The Weyl-Wigner transform^20^ can transform any negative-minimum PDF into a density matrix, which can then always be represented as a complex vector state. The use of neuron modeling obtained^21^ that the successful outcome prediction in a neuron (or small ensemble of neurons) improves the access to nutrients for the neuron. Thus, the continuation of the neuron’s survival requires an ongoing successful broadcast of the validity of the neuron’s predictions. The ontic/existence of the neuron hence requires the success obtained by its epistemic/broadcasting aspect, implying that the requirements for the neuron’s survival are epiontic^4^. The neuronal predictions being statistical as neurons can be perceived as being statistical assessment devices constituted by small ensembles of neurons that are representing information through negative-minimum PDFs^22,23^. Thus, as negative-minimum PDFs are mathematically equivalent to a density matrix which are mathematically equivalent to a vector state, hence the information contained within small ensembles of neurons is mathematically equivalent to a vector state.

In the ECT proposed, the epiontic nature of a system obtains the state extinction from the maximization of the information dissemination, with the implication that during the extinction transition the amplitude of the complex variable representing the vector state associated to the relation between the neuronal ensemble and its environment needs to remain unchanged^4^. This amplitude unchangingness implying that after extinction the system is fully represented by a phase, which agrees with experimental data that the transition between unconscious and conscious states is represented simply by a phase^24^. Thus, the ECT approach explains the existence of a phase-only relation between the neuronal ensemble systems associated to unconscious vector states vs. the ensemble systems associated to conscious vector states. Hence, explaining how neuronal ensemble environments help/create/enhance the preservation of the vector state representing the conscious decision. This phase-only relation explained by the ECT also helps/creates/enhances the corresponding extinction of the vector states representing the unconscious decisions^4^.

The ECT explains the lack of both ignition and posterior cortex synchronization by proposing that consciousness is not induced by these mechanisms, but by the broadcasting of information within the brain with such broadcasting being not just epistemic (as occurs in GWT broadcasting)^19^ but also ontic (as occurs in IIT states)^19^. For the ECT to correctly explain the experimental data of ref^3^. it needs to be possible to have transport of consciousness-related information by WM going from brain regions associated to GWT to those associated to IIT, such a WM connection is most likely the Superior Longitudinal Fasciculus (SLF) as it is that connection that can join the GWT and IIT areas (see Fig. 1). However, for consciousness-related information to be transported across WM the brain’s topology must have the capacity to represent both layers and layer-connecting inter-edges^25^, hence needing for WM connections between the brain regions identified in ref^3^. as being associated to GWT or IIT to be necessarily made of grids having either 2 or 3 axes.

In QD (e.g.)^16^, a vector state of a system is the full describer of that system, likewise, for ECT a vector state in the brain is the full describer of a system represented in a brain thought^4^. Prior to the choice of a system state occurring by the interaction with the environment, the vector states representing a system have a certain ontic aspect to them as the vector states are all that the system is before the measurement (meaning that they are all that can be said about the system); and after the measurement, the vector states of the system have a certain epistemic aspect to them as the system does not completely adopt an objective existence but only does so in as much as it is capable of leaving imprints about itself in the environment. Hence, these vector states are called epiontic. A consequence of the vector states being epiontic is that it makes both the system vector state basis and the environment vector state basis become orthogonal^16^. It has been proposed that also for the case of system representations within the brain, the vector associated to a represented system are epiontic^4^, meaning that the information at a certain representation-level in the brain is introspectively focused as it only relates to other information in the brain at that same representation-level.

### Improvement of diffusion MRI tractography by use of gridography

**Fig. 2:**
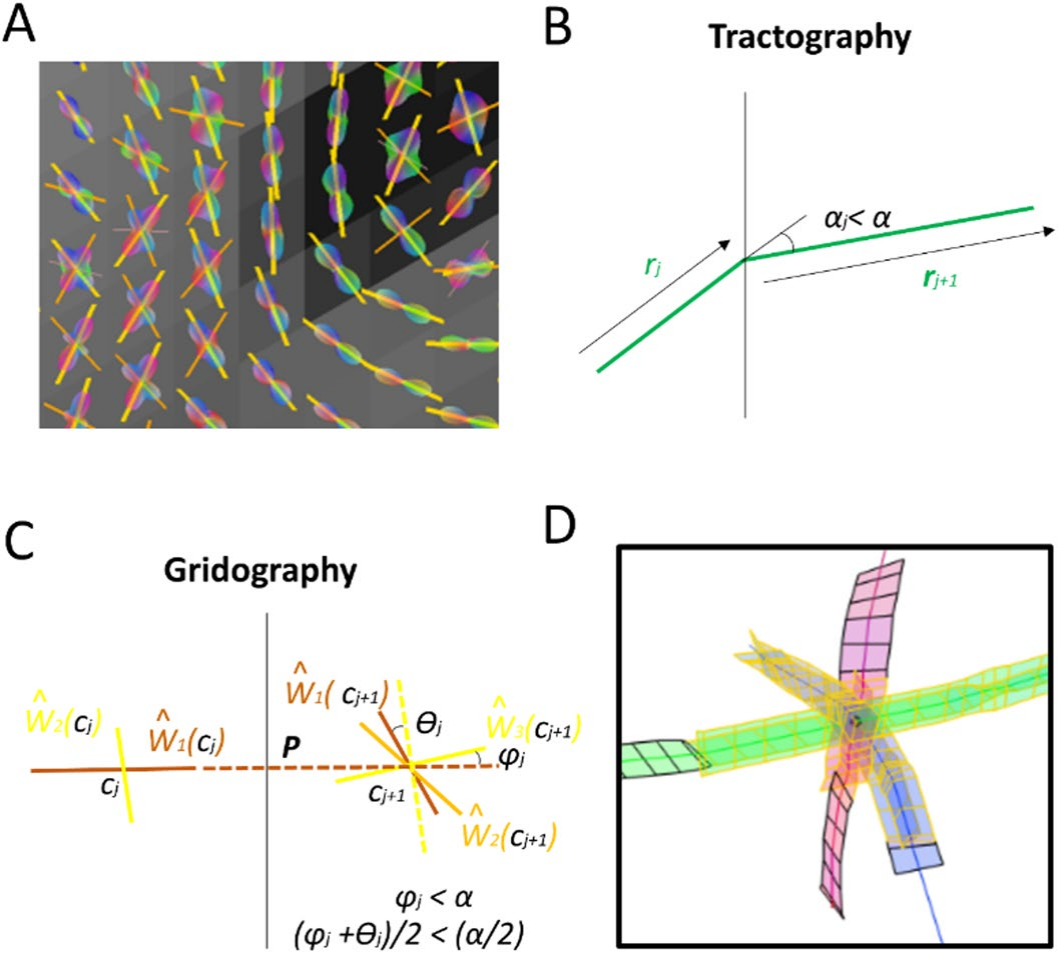
Description of information represented in gridography that usual tractography does not address. **(A)** 3D-plot of anisotropic component of ODF and axes in WM region overlaid over anatomical images, using DSI data from MGH-UCLA branch of HCP. Coloration of directions of ODF peaks: red, x-axis; green, y-axis; blue, z-axis. Coloration of axes: Yellow, main axis (highest value of ODF); Orange, secondary axis; Brown, tertiary axis. **(B)** Tractography lines expressed in green occur if a step **r**_j_ at a certain location has a α_j_ orientation difference to the next step **r**_j+1_ that is less than a threshold α. **(C)** Gridography expressed in brown occur if a grid is propagated along each one of its axes which are always 0 to 3 axes, with gridography advancement occurring only if for the director-axis **P** (here an extension of **ŵ**_**1**_**(c**_*j*_**)**) the most similar axis in the next location (here **ŵ**_**3**_**(c**_*j+1*_**)**) has an angle difference with the director-axis (here **φ**_*j*_) that is less than α, and the average of the angles between the axes ensemble of the two different locations (here **φ**_*j*_ and **θ**_*j*_) average to less than α/2. Coloration of axes: Yellow, main axis (highest value of ODF); Orange, secondary axis; Brown, tertiary axis. **(D)** Example of gridography result with one seed. Each seed point gets a maximum of 6 possible directions, since each voxel has no more than 3 axes, and each axis has two opposite directions. For 2-axes cases the edges are black, and for 3-axes cases the edges are yellow.

At the start of the use of diffusion Magnetic Resonance Imaging (MRI) tractography^27^, the processing methodology was based on Diffusion Tensor Imaging (DTI)^27^, which evolved in data processing + acquisition complexity towards Q-Ball Imaging (QBI)^28^ and Diffusion Spectrum Imaging (DSI)^29,30^. In the Human Connectome Project (HCP), three technologies are combined: diffusion MRI, default-state functional MRI, and genomics. The diffusion MRI data of the MGH-UCLA branch of the HCP project was used to test the gridography approach^31^, as such data is extremely high-quality Cubic Acquisition Diffusion Imaging (CADI) data with similar structure to that used in ref^32^. The tractography usually represents its results by either lines conduced along WM axes^27,30^ or by probabilistic maps based on the WM axes^33^, whereas gridography approach^31^ (see Fig. 2) is based on the orientation similarity of neighboring WM axes ensembles. In gridography, instead of usual tractography’s line orientation comparison for neighboring locations, it is the local axes systems’ orientational arrangement which are compared across locations.

The simplest tractography approach is the deterministic approach to construct WM lines using DTI^27^; in which the track steps building the WM lines are determined at each WM brain location in a unique fashion by an axis **ŵ**_*1*_. For the case of crossing WM fibers identified using tractography, at each WM brain location there are $\:k$ axes **ŵ**_*n*_ with *n* being an integer between 1 and $\:k$, and along each of these axis passes a WM fiber bundle. There are two major approaches to data processing of high-resolution diffusion MRI data: either deterministic (e.g. using deterministic Trackable Directions (dTD))^34^, or probabilistic (e.g. using probabilistic Trackable Directions (pTD))^34^. For the dTD approach, for each of the **ŵ**_*n*_ axes a WM track line passes along it, provided that for such axis there is an axis in a neighboring location that is within an angle threshold of $\:\propto\:$. For the pTD approach, each of the axis’s orientations has an associated probability and so the resulting WM tractography maps are probabilistic. The gridography approach is different from both the dTD and the pTD approaches, in that the $\:k$ axes systems in neighboring locations are connected if for each of the axes **ŵ**_*n*_ there is an axis in a neighboring location which is within an angle difference so that the average of that difference for the different axes is below $\:\propto\:/2$. The dTD and pTD approaches appear in ref^34^. as representing generalizations to the most common approaches to tractography, which indeed they are, as almost all tractography approaches except for gridography can be divided between DTI, dTD and pDT, with gridography being an axes ensemble-driven dTD instead of the usual multiple axis-driven dTD.

In gridography, we use the highest diffusion orientation of the 3 highest ODF directions obtained previously (see Fig. 2A) where the axis extension and parallelism detection are only performed for WM voxels, and we choose an angle threshold $\:{\upalpha\:}={30}^{o}$ as it is considerably smaller than $\:{45}^{o}\:$without being too small. This implementation consists in starting at a seed point in WM called the central voxel, $\:{\mathbf{r}}_{0}={\mathbf{c}}_{0}$ and going in the 6 directions defined by the 3 vector axes **ŵ**_*n*_(**c**_*o*_) constituting the columns of $\:\widehat{\mathbf{W}}\left({\mathbf{c}}_{0}\right)$. In the *j*-th iteration, *j>0*, the point $\:{\mathbf{c}}_{j}$ is located within a voxel with 3 vector axes expressed in matrix $\:\widehat{\mathbf{W}}\left({\mathbf{c}}_{j}\right)$, with a director-axis starting from $\:{\mathbf{c}}_{j}$ going to the boundary of that voxel by addition of vector $\:\mathbf{P}$ which is the director-axis vector that links a voxel to the neighboring voxel, with $\:\mathbf{P}$ being parallel to one of the axes at $\:{\mathbf{c}}_{j}$, the next step hence being: $\:{\mathbf{c}}_{j+1}={\mathbf{c}}_{j}+\mathbf{P}$ (see Fig. 2C). At that next point $\:{\boldsymbol{c}}_{j+1}$, a comparison is made between the 3-axes ensemble at $\:{\boldsymbol{c}}_{j+1}$ and the 3-axes ensemble at $\:{\boldsymbol{c}}_{j}$, for each axis that is similar for both locations a value of 1 is added to $\:k$. Thus, for the same voxel different values of $\:k$ can occur. The gridography number of axes in the ensemble is denoted by $\:k\left(\widehat{\mathbf{W}}\left({\mathbf{c}}_{j}\right),\widehat{\mathbf{W}}\left({\mathbf{c}}_{j+1}\right),\mathbf{P},{\upalpha\:}\right)$ (see Eq. 2 of Methods section), where the $\:\widehat{\mathbf{W}}\left({\mathbf{c}}_{j}\right)$ and $\:\widehat{\mathbf{W}}\left({\mathbf{c}}_{j+1}\right)$ are respectively associated to the coordinates $\:{\mathbf{c}}_{j}$ and $\:{\mathbf{c}}_{j+1}$. Meaning that for each $\:\widehat{\mathbf{W}}\left({\mathbf{c}}_{j}\right)$ matrix, the $\:\widehat{\mathbf{W}}\left({\mathbf{c}}_{j+1}\right)$ matrices are associated to axes located in one of the 26 voxels neighboring the voxel where the point associated to the matrix $\:\widehat{\mathbf{W}}\left({\mathbf{c}}_{j}\right)$ is located (only the WM-located $\:\widehat{\mathbf{W}}\left({\mathbf{c}}_{j}\right)$ and $\:\widehat{\mathbf{W}}\left({\mathbf{c}}_{j+1}\right)$ matrices are considered). The two axes at different neighboring voxels along a director-axis $\:\mathbf{P}$ are considered parallel when the angle between them is less than $\:{\upalpha\:}$, and the axes different from $\:\mathbf{P}$ are considered parallel (hence connected) if the average angle difference between somewhat parallel axes localized at the two extremities of $\:\mathbf{P}$ is less than $\:{\upalpha\:}/2$. If for a certain voxel, the number of given axes is less than 3, then, $\:\widehat{\mathbf{W}}\left({\mathbf{c}}_{j}\right)$ or $\:\widehat{\mathbf{W}}\left({\mathbf{c}}_{j+1}\right)$ contain zero columns, for the size of these matrices must be 3 × 3.

The DSI data processing work of ref^35^. obtained that the brain’s WM structure has a 3-axes structure local topology, meaning that it is extremely rare for the number of axes to be higher than 3. The QBI-based processing of High Angular Resolution Diffusion Imaging (HARDI) data of WU-Minn-Ox branch of HCP which was presented in ref^34^. obtains that it is highly unlikely that the number of axes is higher than 3. Moreover, gridography results using the MGH-UCLA branch of HCP data^31^ also obtained that usually, at most, $\:k=3$. It is analyzed in this work whether the $\:k=2$ or $\:k=3$ gridography regions are indeed the WM connections joining the regions identified in ref^3^., hence justifying the integration of GWT with IIT proposed by the ECT approach.

### Expected gridography results based on consciousness theory

The major differences between the different ToCs consist in^19^: i. a focus on global vs. local states, ii. a focus on phenomenal vs. functional properties, iii. brain location of their neuronal correlates being anterior vs. posterior. The HOT is mostly local + phenomenal + anterior, the GWT is mostly global + functional + anterior, the IIT is mostly “global & local”+phenomenal + posterior, whereas the PPT is mostly “local & global”+”functional & phenomenal+”anterior & posterior”. The PPT approach expresses a general statistical approach to the neural correlates of consciousness, with such a statistical approach being again assessed in future paragraphs, with the relevance of the predictive processes in PPT playing a similar role to the validity of the meta-representations in the HOT approach^19^. Moreover, as the brain locations of HOT and PPT approaches are very similar, it can be inferred that the PPT approach simply describes a statistical approach to information processing in the brain, thus clarifying the information-flow structure of both the HOT and GWT approaches.

The HOT and GWT mostly differ by the inclusion of the need for an ignition and broadcast events within GWT, the PPT and HOT and GWT approaches are hence mutually compatible, being thus possible to consider a HOT + GWT + PPT approach that can then be compared with the IIT approach^19^. However, relative to GWT, the IIT approach says little about how consciousness compares with other aspects of the mind such as attention, learning and memory^19^; therefore, a certain extension of IIT needs to be attempted. Indeed, such endeavors have been made within IIT by making assessments of shared information between agent and environment, but that is what PPT tries to achieve, so it can be said that what is being advanced is IIT + PP. Thus, the two contemporary competing approaches are HOT + GWT + PPT and IIT + PPT approaches, which we henceforth respectively call the GWT* and IIT* approaches. Hence, for GWT* consciousness is mostly anterior, whereas for IIT* consciousness is mostly posterior^19^.

In the PPT approach, it is highlighted the role of neuronal ensembles as statistical estimators^19^, which thus result in the GWT* and IIT* approaches. It is accepted in the neuroscience literature that neurons can be perceived as statistical assessment devices, as small ensembles of neurons are known to represent negative-minimum PDFs^22,23^; it is thus not a problem for neuronal ensembles to represent statistical PDFs. Looking at Fig. 1 which includes a condensed form representation of contemporary neuroscience’s assessment of the relation between brain function and anatomical location^4^, it can be perceived that GWT* perceives consciousness as being about the Out-Then whereas IIT* perceives consciousness as being about the In + Now (see Fig. 1 for Out-Then and In-Now brain area locations); for GWT* is more about functional processes and hence more epistemic, whereas IIT* is more about beingness phenomena and hence more ontic.

Recent work compared the experimental validity of GWT vs. IIT approaches^3^, where it was found that both were partially unsatisfactory, with GWT being challenged by the lack of occurrence of an ignition at stimulus offset and IIT being challenged by a lack of sustained synchronization within the posterior cortex. Although both GWT and IIT are challenged in the experimental results of ref^3^., it is mostly GWT that is challenged with IIT being mostly vindicated. Thus, from the lack of complete match with either GWT or IIT it can be inferred that: (i) consciousness is not induced by synchronization but by broadcasting of information, (ii) ignition does not occur at stimulus offset but it is the broadcast alone that induces consciousness, (iii) as neither GWT nor IIT are in total agreement, a flow of consciousness-related information moving across WM must be possible to occur which requires a WM connection with specific characteristics.

What has not been experimentally vindicated in IIT^3^ is the idea that consciousness arises by synchronization in the posterior regions, but rather it seems that it is the broadcasting of information that allows for the occurrence of consciousness. Thus, it is the broadcasting of irreducible maxima of integrated information that allows for consciousness to occur. Hence, it is the transport of consciousness-related information from the In + Now anterior region to the Out + Then posterior region that allows for consciousness to occur (see Fig. 1 for the In + Now and Out + Then brain locations). Such information must be carried by WM with the adequate structure, but to assess what that structure needs to be, it is necessary to analyze how consciousness-related information can be mathematically represented, and for that we use a recent mathematical approach described in ref^25^. which proposes that an axiomatic representation of a ToC is based on multilayer networks understood as a graph theory extension of the mathematical substrate of IIT. In Figs. 1 and 2 of ref^25^. it is made clear the role of layers and edges connecting layers for the irreducible representation of consciousness-related information, where layers are 2-dimensional with edge-connection making it 3-dimensional, implying that it expected that the WM structure associated to it has a topology of $\:k=2$ (2 axes) or $\:k=3$ (3 axes).

**Fig. 3:**
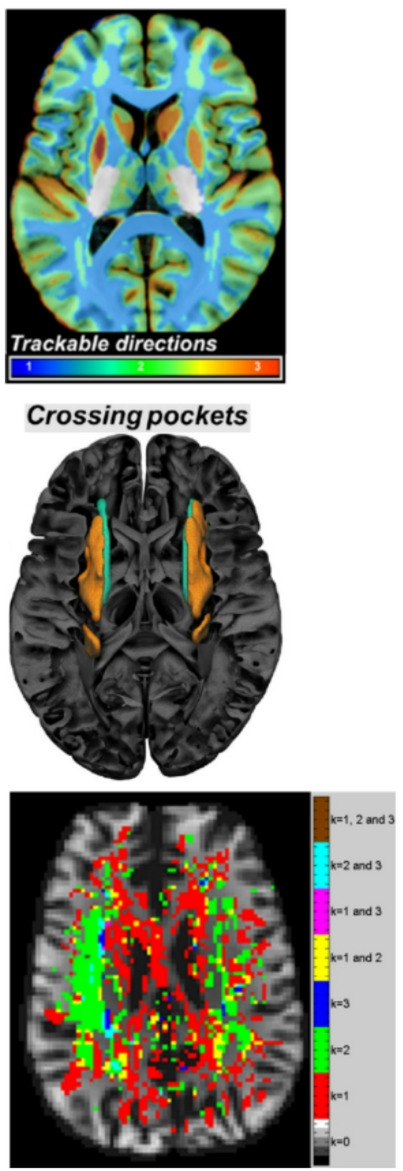
Comparing usual tractography with gridography it is obtained that both methods obtain WM axes in similar number and principal orientation, but gridography provides a new meaning to tractography’s “crossing pockets” thus coherently going more posterior than usual tractography. **Top)** Number of axes directions for each voxel, obtained by tractography based on HARDI data of WU-Minn-Ox branch of HCP in ref^34^., with colors in lower horizontal bar indicating the number of axes. **Center)** “Crossing pockets” represented in 3D perspective over brain anatomy, based on HARDI data of WU-Minn-Ox branch of HCP in ref^34^. **Bottom)** Gridography results using 300 mT/m gradient Siemens MAGNETOM Skyra CONNECTOM 3 T scanner CADI data of MGH-UCLA branch of HCP in ref^31^. : *k = 0* is colored using anatomical MRI, the other values of *k* have colors based on the description in the vertical table on the right.

The in-vivo postsynaptic time histograms (PSTHs) obtained in non-human animals’ cortex best data fit are cliques of neurons representing the basic units of information, with the experimentally found highest dimensionality of cliques obtained in statistically relevant quantities being a clique having 3-simplices (see Fig. 3D (left) and Sect. 4.1.5 of ref^36^. implying that the clique is composed by 4 neurons, meaning it is a 3-axes process which indicates that most of the brain’s “thinking” occurs in 3D; “thinking” which also includes the “thinking about feelings” that was proposed in refs^37,38^. as occurring through 3-axes representations associated to the body’s 3-axes. This best fit to the in-vivo PSTHs obtained in non-human animals’ cortex^36^, also obtains that the neurons implement a classical diffusion event, describable by a classical Continuous Markov Process (CMP), which is capable of explaining in detail the experimental results for rats undergoing decision-making tasks^39^. Using the classical CMP perspective of the Hammersley-Clifford Theorem (HCT), it can be inferred that what the rat’s neurons are doing is to use the HCT to establish a one-to-one relation between the edge-interconnected cliques of neurons (where edges are synaptic connections between neurons, and nodes are neurons’ cell bodies) with the physiological 3D areas constituting the vicinity of the neurons’ cell bodies, meaning its neighboring neurons^40^.

The brain’s WM DSI tractography results of ref^35^. obtained a 3-axes structure, which agrees with the expected gridography results that $\:k$ is at most equal to 3. Moreover, the 3-layers QBI results of ref^34^. expressed in Fig. 3-Top obtain that $\:k$ is at most equal to 3. The anatomical WM regions identified as being a challenge to tractography algorithms due to abundance of crossing fibers and a low Generalized Fractional Anisotropy (GFA) (a scalar indicating the average variance of the Orientation Distribution Function (ODF) amplitude for different gradient orientations), were indicated in the results of ref^34^. and are expressed in Fig. 3-Center by orange “crossing pocket” WM regions located dorso-laterally to the genu of the corpus callosum and extending in the posterior direction. Thus, the “crossing pocket“ regions correspond to the anterior-to-middle portions of the sections I and II of the SLF as expressed in ref^26^., where the “crossing pocket” region is signaled in ref^34^. as a large continuous region with a high number of WM fiber crossing (see Fig. 3-Center). The use of HCP data in gridography^31^, as the Fig. 3-Bottom CADI diffusion MRI results indicate, obtains gridography results going more anteriorly than the results of Fig. 3-Center but with number of gridography axes per voxel like the number of axes per voxel of Fig. 3-Top.

## Results

An example of anisotropic components of the ODF with corresponding fiber orientations is displayed in Fig. 2A. The relative angles between fiber directions at different locations that allow constructing the gridography are more elaborate than the more common DTI/dTD approaches described in Fig. 2B and appear in Fig. 2C. The gridography is generated by using the local axes to define 3 × 3 matrices at each WM voxel. From those matrices, for each pair of voxels $\:k$ compatible axis orientations are defined, a $\:k$ for each pair. An example of a single seed-point gridography result appears in Fig. 2C; the $\:k$=2 always have black edges, and the $\:k$=3 always have yellow edges. For each gridography “line” such as the displayed in Fig. 2C, at each step there corresponds a $\:k$ value; but a different gridography “line” might have a different value of $\:k$ for the same voxel.

**Fig. 4:**
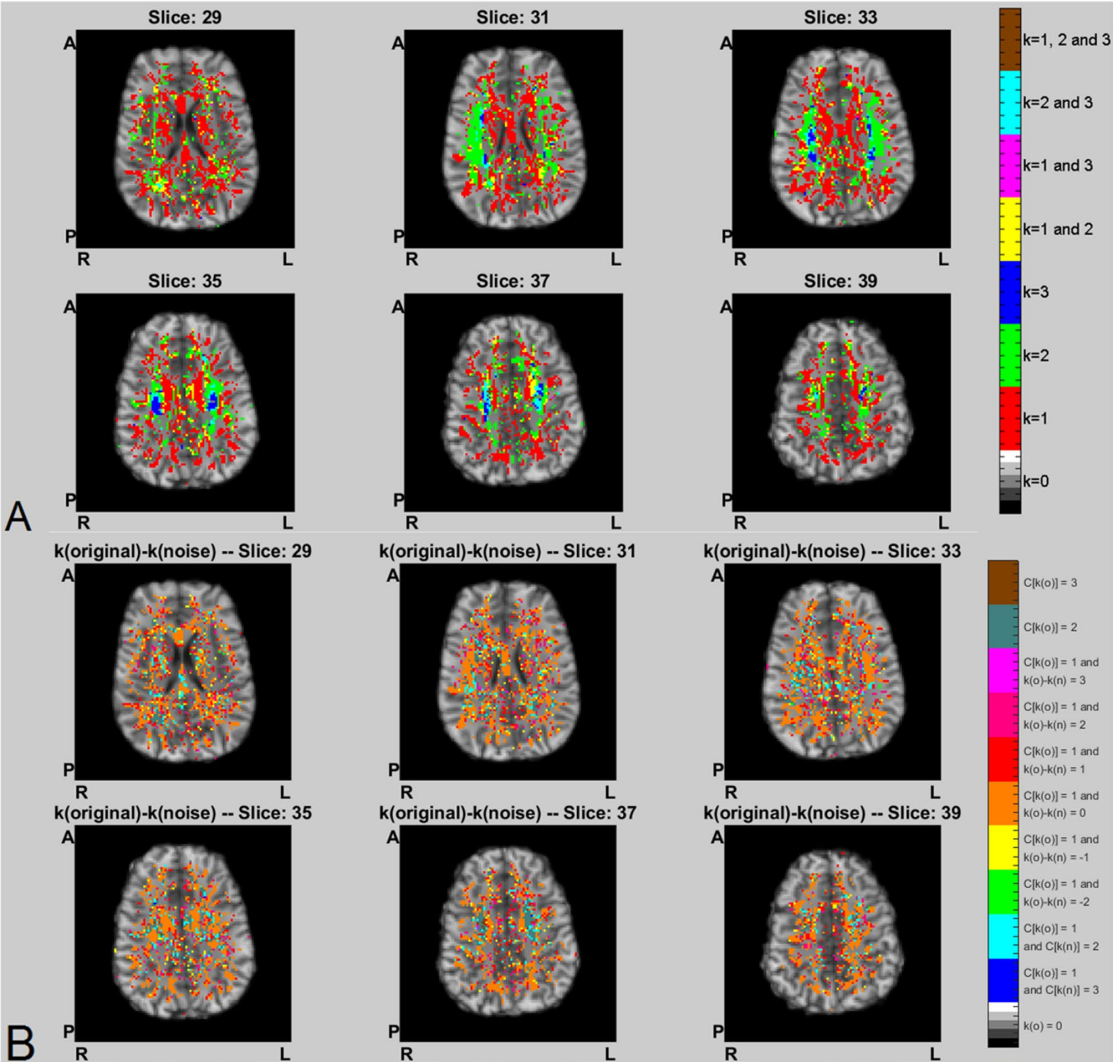
WM location of gridography results plus the verification of their strong noise-insensitivity. **(A)** RGB color maps of the values of *k* in WM voxels, overlaid on an anatomical MRI. Color codification is described in corresponding vertical bar, when the value is zero the displayed gray scale is from the anatomical MRI using MGH-UCLA HCP data DSI results overlaid on MGH-UCLA HCP anatomical MRI. **(B)** RGB color maps of the influence of noise over the values of *k* in WM voxels obtained using DSI data processing of MGH-UCLA HCP branch data, overlaid on an anatomical MRI. The *k*(original) denotes the value of *k* obtained using original data, and the *k*(noise) denotes the value of *k* obtained using data with added noise. Color codification is described in corresponding vertical bar, when the value is zero the displayed gray scale is from the anatomical MRI; with the $\:C\left[\right]$ symbol meaning the count of different values of *k* within a voxel’s vicinity, whereas *k*(o) means *k*(original) and *k*(n) means *k*(noise).

Figure 4A displays the WM RGB color maps of the $\:k$ values between the central voxel and neighboring voxels, as obtained by gridography, using MRI data from the MGH-UCLA branch of HCP. The WM RGB color maps of the influence of noise over the values of *k* for each voxel (Fig. 4B), obtain that for the MGH-UCLA HCP data the noise for most voxels is not strong enough to affect the value of *k*. Noise was added as described in eq. (A1) of ref^41^. We tested the effect of noise by adding noise to the original MGH-UCLA HCP data, and then calculated the k by the same process as for the original data. These results only analyze a sub-portion of the possible trajectories between voxels but were not significantly affected when the number of possible trajectories is increased, and thus this indicates that the noise does not significantly affect the gridography results for the “no k change” WM regions in orange areas of Figs. 3B.

**Fig. 5:**
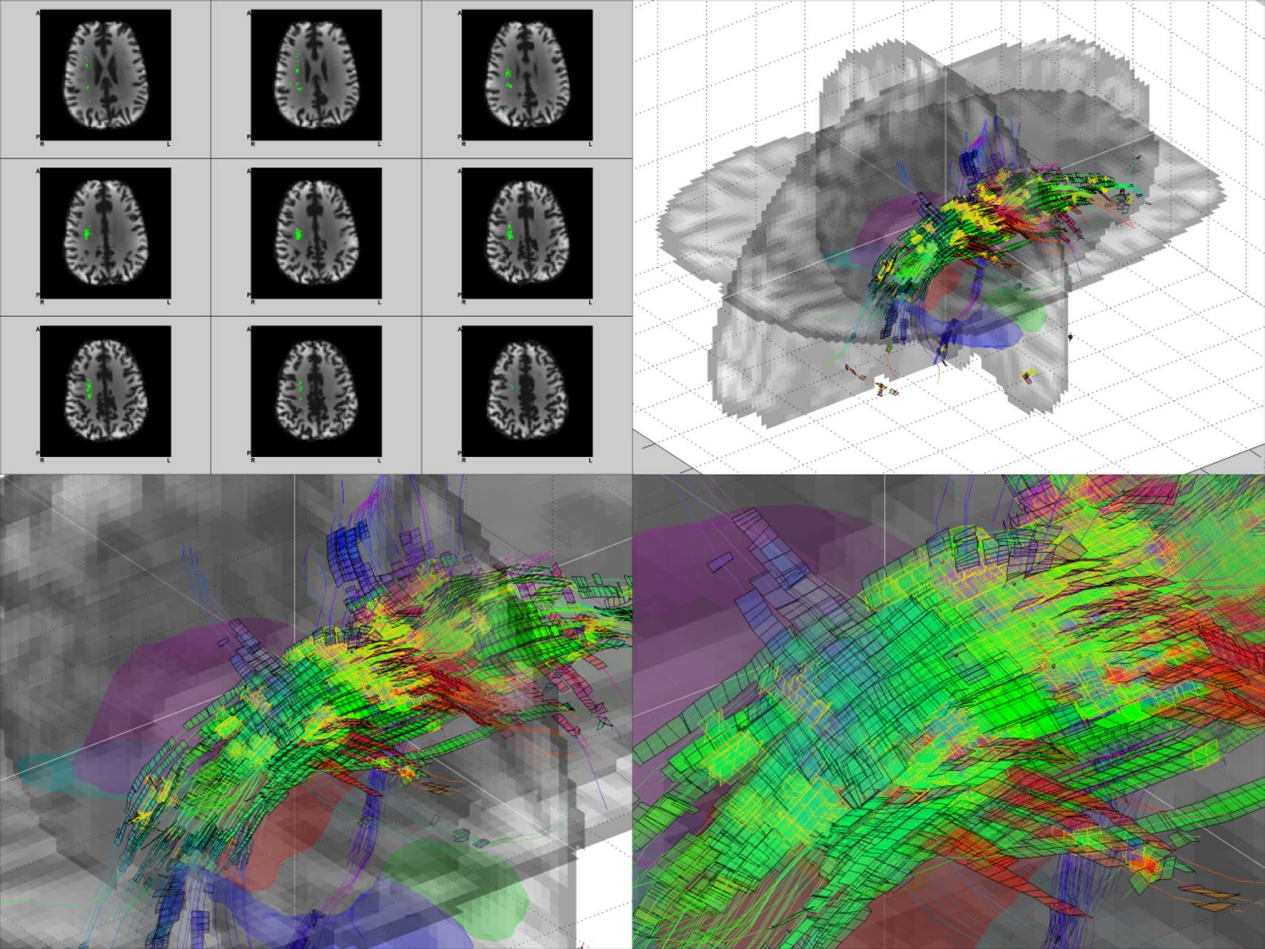
3D gridography results where the occurrence of different types of structure can be observed. **Upper left)** Region-Of-Interest (ROI) in RH marked by green, corresponding to the regions where noise mostly does not change the value of *k* which is 3, overlaid to a MGH-UCLA HCP anatomical MRI of the corresponding slice. **Upper right)** 3D Plot of gridography results, performed in WM regions, overlaid by 3 crossing translucent anatomical images, using DSI data from MGH-UCLA HCP. The seed points are marked by brown circles at the center of those voxels. The regions of seed are similar as ROI obtained in upper left sub-image but applied not only in presented transversal slices but also in remaining transversal slices. The white lines are the intersection of anatomical images. Coloration of anatomical surfaces: Yellow, Left Amygdala; Green, Right Amygdala; Cyan, Left Hippocampus; Blue, Right Hippocampus; Magenta, Left Thalamus; Red, Right Thalamus. Coloration of lines and planes orientations is: red, x-axis; green, y-axis; blue, z-axis. Coloration of plane edges is: Black, one-plane containing the propagation line; Yellow, two-plane grid intersecting at the propagation line. **Lower left)** Amplification by 2.3 of upper right image. **Lower right)** Amplification by 2.3 of lower left image.

**Fig. 6:**
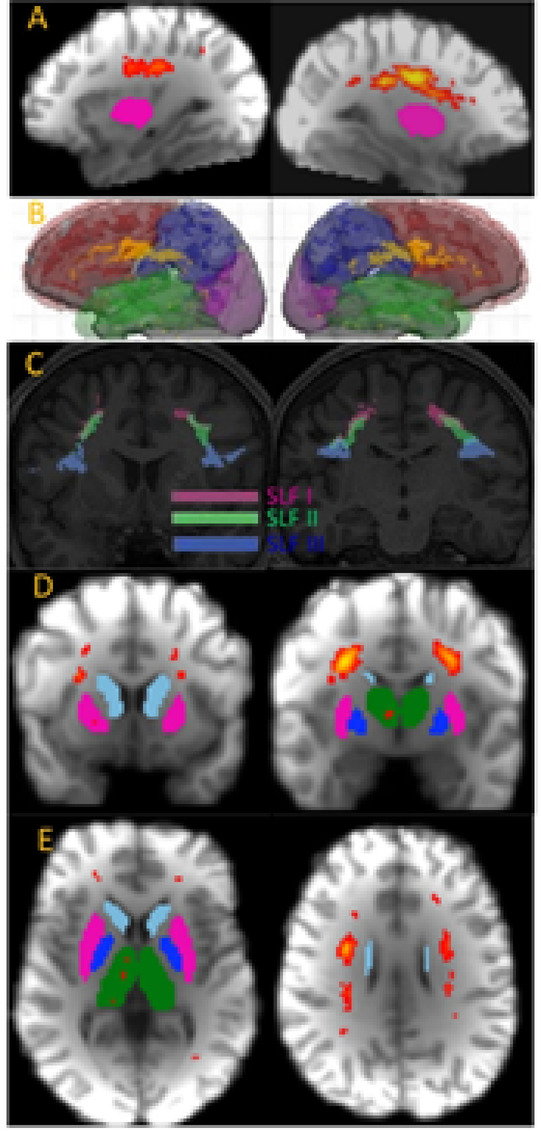
Location of gridography results is in the Superior Longitudinal Fasciculus (SLF), specifically in sections I and I but not in section III. **(A)** 2D visualization of the $\:k$*=3* histogram, in a red-yellow scale, overlaying sagital anatomical images (left image is left hemisphere, right image is right hemisphere) with segmented regions. The segmented regions are the Caudate (light blue), Globus Pallidus (dark blue), Putamen (magenta), and Thalamus (green). **(B)** 3D Plot of gridography results, showing only the yellow edges of two-plane grid with *k = 3*, overlaid by translucent anatomical surfaces. The opaque gray surface is the separation wall between the lateral component and the medial component of hemisphere (left image is left hemisphere, right image is right hemisphere). Coloration of anatomical surfaces for both images is Red for Frontal Lobe, Green for Temporal Lobe, Purple for Occipital Lobe, and Blue for Parietal Lobe. **(C)** Anatomical location of SLF in coronal view; with separation between sections I, II and III (from ref^26^. **(D)** Same representation as A) but from a coronal view at about the same slice as the corresponding image in C) just above it, with left side of each image being the left hemisphere. **(E)** Same representation as A) but from a transverse view with left side of each image being the left hemisphere.

**Fig. 7:**
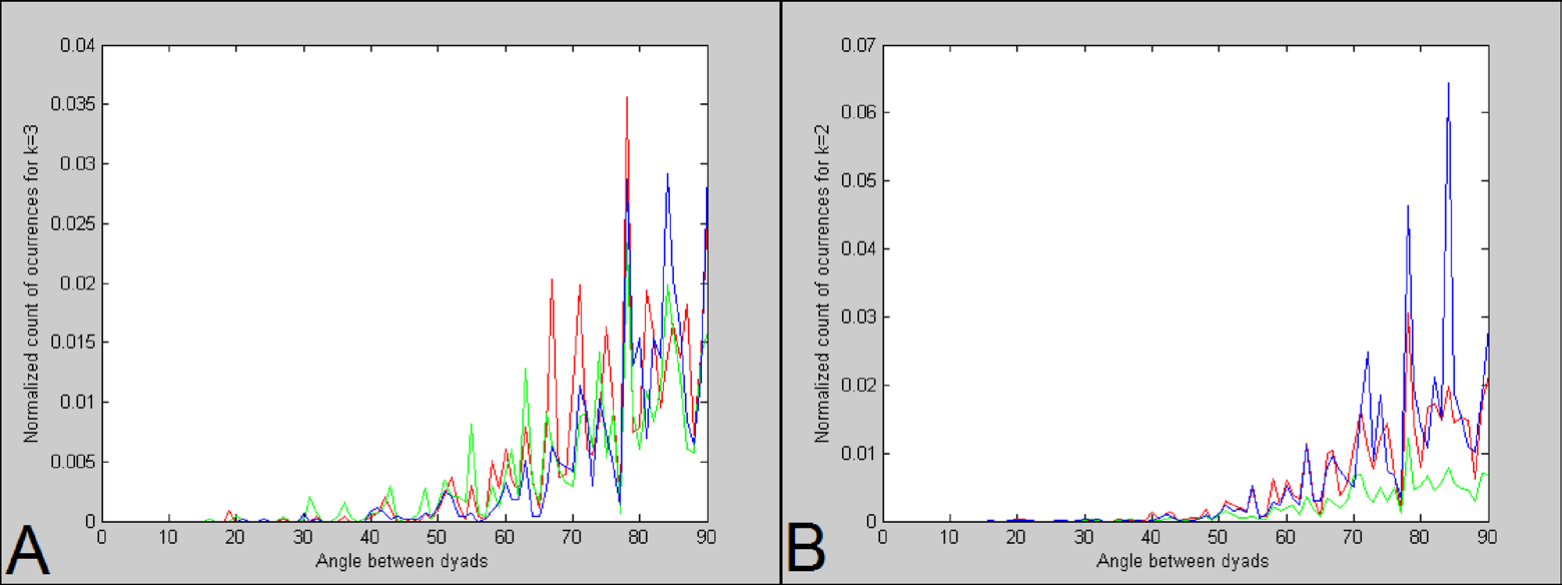
Angle between pairs of possible selected axes versus count of occurrences normalized by the total number of occurrences is obtained to be mostly orthogonal. Data are presented for both: **(A)** $\:k$=3, and **(B)** $\:k$=2. The red, green and blue lines correspond to the count of occurrences of angles between pairs of selected axes defining a plane whose orthogonal is closest to, respectively, the brain’s anatomical x-axis (red), y-axis (green), and z-axis (blue).

The seed points for the gridography results in Figs. 5, 6 and 7 are located at the center of those “no k change” voxels. We used those seeding points, mainly localized in the SLF of the right hemisphere (RH) in Fig. 5, where the yellow edges correspond to $\:k$=3. For $\:k$=3 there are 3 axes, respectively the propagation line axis and the two crossing WM fiber axes for each of the gridography “line” steps. In Fig. 6B, we made the brain translucent and inserted an opaque grey sagittal slice dividing the medial from the lateral section of each of the hemispheres; the yellow corresponds to $\:k$=3 areas and are found mainly in the Frontal and Parietal Lobes.

By counting the number of $\:k$=2 or $\:k$=3 relations between voxels, we obtained a histogram of the number of relations for each voxel. These results are shown in Fig. 6A, D and E. As expected, there are more voxels with $\:k$=2 relations than $\:k$=3 relations.

The $\:k$=1 and $\:k$=2 and $\:k$=3 map in Fig. 4A has anatomical location similarities to the 1-directon and 2-direction and 3-direction map in both Figs. 1 and 2 of ref^34^., but in this work the use of gridography instead of tractography allows the results in Fig. 4A to identify a grid topology that tractography does not aim to identify, with such gridography topology joining tractography lines with 2-planes gridography when $\:k$=2, and 3-planes gridography when $\:k$=3 as represented in both Figs. 2C and 5. In Fig. 5, it is of notice that although the anatomical location of the regions identified using gridography are similar to the “crossing pockets” defined in ref^34^. and represented in Fig. 3-Center, the more posterior regions of the gridography results of Fig. 5 do not appear in those “crossing pockets” results, with those more posterior regions of the gridography results having both $\:k$=2, and $\:k$=3 locations but with $\:k$=2 being more prevalent. The regions with higher count for both $\:k$=3 (Fig. 6) and $\:k$=2 (data not shown) are located in the SLF (sections I and II, but not section III; see Fig. 5C). In Fig. 6, we also observe some counts in the crossing of the forceps minor and the anterior thalamic radiation; with the counts in the forceps minor and anterior thalamic radiation being in good agreement with the location of the “crossing pockets” identified in ref^34^. (see Fig. 3-Center), but as our identified regions extend more posteriorly they are able to explain an interaction between GWT and IIT activated regions, which the results in ref^34^. could not. The almost orthogonality of the results in Fig. 7 agree well with the almost orthogonality of the topological results identified in ref^35^. meaning that most angles between axes are less than 10º from 90º angles, and where the topological structures in gridography being identified automatically.

## Discussion

The recent experimental results of the neuroscientific study of consciousness^3^ obtain that a certain WM connection between anterior and posterior portions of the brain is required to have a certain type of topological infrastructure, with the form of WM tractography described in this work, called gridography, being able to obtain that such WM connection with the appropriate topology does exist. The existence of such WM connection with appropriate topology helps allow the possible validity of the ECT approach to consciousness described in ref^4^. and in this work. More specifically, the results predicted in Fig. 1 were observed in Figs. 4, 5 and 6. Moreover, the expected orthogonality of the WM structure associated to consciousness^4^ due to the phase-only relation between neuronal ensembles associated to unconscious states vs. those associated to conscious states, was partially obtained as described in Fig. 7. The MGH-UCLA dataset used for gridography is a small dataset, so in the future a further analysis should be provided by increasing the number of subjects and by testing different magnetic field gradient intensities.

Hala Point implements approximately 1.15 billion neuromorphic neurons interconnected by 128 billion synapses, which facilitate bottom-up approaches in AI ethics. Although Hala Point’s processing power is still far behind that of a human brain, which has around 86 billion neurons, the increasing sophistication of such systems raises questions about the potential for AI consciousness. Furthermore, considering that intellectual capacity across animal species is influenced by brain-to-body ratio, it is plausible that developing consciousness in AI systems would also necessitate interaction with a body or a sensory robot which replicates the body/robot topology within the AI’s structural framework, akin to the integration observed in human WM topology. As it is important to design AI systems that can operate ethically even when developers’ skills or oversight may be limited; and given that similarity between AI systems and biological brains has also grown as exemplified by the development of large-scale neuromorphic systems like Intel’s Hala Point, which uses Loihi 2 processors^5^; thus, a better understanding of how consciousness arises in humans is likely to be key for the development of more ethically adequate AI.

## Methods

We used CADI data available online obtained in the MGH-UCLA branch of HCP from a Siemens MAGNETOM Skyra CONNECTOM 3 T scanner with a maximum gradient amplitude of 300 mT/m, which has 514 gradient directions, with an image size of 104 × 104, and 55 transversal slices. The used CADI data is similar to the DSI data of ref^32^. and is constituted by 2 normal subjects (presented data is from one of the subjects, but the other subject has similar results). We used TrackVis software to do DSI processing of this CADI data to obtain a 181 points ODF surface. The obtained ODF data contains both non-normalized ODF values and ODF peaks (e.g. Figure 2A). If the obtained ODF contains more than 3 pairs of peaks, we only use the highest ODF 3 pairs of opposing peaks to perform the gridography. Furthermore, we also used the anatomical labeling provided by FSL; so that WM can be distinguished from gray matter (GM), cerebro-spinal-fluid (CSF), and everything else not WM. The anatomical T1 image was inhomogeneity corrected, contrast adjusted, voxel re-sampled, and co-registered to the diffusion MRI data. Results obtained for lower gradient strength CADI diffusion MRI data were found to be of too low quality for analysis and so that data was not used.

The gridography method consists in starting at a seed point in WM called the central voxel, **r**₀=**c**₀ and going in the 6 directions defined by the 3 vector directions as columns of **Ŵ**(**c**₀). In the *j*-th iteration, *j* > 0, the point **r**_*j*_ is always at a voxel boundary and it follows an orientation **t̂**_*j*_. The next point is determined by the vectorial equation: **r**_*j*+1_=**r**_*j*_+*h*_*j*+1_**t̂**_*j*+1_, where *h*_*j*+1_ is the positive length of the limited by the border of a voxel with center at **c**_*j*+1_, in the (*j* + 1)th iteration. We assume that the value of *h*_*j*+1_ is positive, so that when a trajectory hits the border of a voxel it will always exit that voxel.

The **t̂**_*j*+1_ is determined from the matrix **Ŵ**(**c**_*j*+1_), where **c**_*j*+1_=round(**r**_*j*_+$\:\sqrt{{\epsilon}_{M}}\:$**t̂**_*j*_)) and round(**x**) returns a vector whose elements are the rounded elements of **x**; whereas *∈*_*M*_=2.2*10⁻¹⁶ is the machine precision. The **t̂**_*j*+1_ is obtained by selecting the column of **Ŵ**(**c**_*j*+1_) with the lowest angle between itself and **t̂**_*j*_, provided this minimum angle is less than α; and then multiplying the selected column by the signal of the dot product between this column and **t̂**_*j*_, so that the line propagation is not allowed to go back. If there are 2 or 3 columns of **Ŵ**(**c**_*j*+1_) with the same minimum angle, the column with highest value of ODF is selected. The **r**_*j*+1_ is defined to be long enough for its end-point to be located in the voxel’s boundary, the value of *h*_*j*+1_ is then computed, by resolving the cube surface equation, where ||**r**||_+∞_=max(|r_x_|,|r_y_|,|r_z_|) is the infinite-order norm of **r**:

1 $$\:{||{\mathbf{r}}_{j}+{h}_{j+1}{\widehat{\mathbf{t}}}_{j+1}-{\mathbf{c}}_{j+1}||}_{+{\infty\:}}=\frac{1}{2}$$

After this, a line is traced from **r**_*j*_ to **r**_*j*+1_ and colored according to the direction of **t̂**_*j*+1_ divided by the infinite-order norm. The iterative algorithm stops if either the point is outside of WM, the point is outside the image, all the axes systems of the current voxel are zero, the minimum angle between **t̂**_*j*_ and **t̂**_*j* + 1_ is greater than α, none of the values of *h*_*j*+1_ is positive, the propagation line is in the voxel boundary, or *k*_*j*_ = 0 (see Eq. (5)). Then, the result of tracing lines for all iterations is a piecewise line. Additionally, in order to draw topological structures, we compute the value of *k*_*j*_ from equation below, where $\:{\mathbf{P}}_{j}=({\mathbf{r}}_{j+1}-2{\mathbf{r}}_{j}+{\mathbf{r}}_{j-1})/\parallel{\mathbf{r}}_{j+1}-2{\mathbf{r}}_{j}+{\mathbf{r}}_{j-1}\parallel$, and $\:\mathcal{K}$ is the is the number of axes for which the angles $\:{\widehat{\mathbf{W}}\left({\mathbf{c}}_{j}\right)}^{\boldsymbol{T}}\widehat{\mathbf{W}}\left({\mathbf{c}}_{j+1}\right)$ are less than α for axes along $\:{\mathbf{P}}_{j}$ and less than α for axes orthogonal to $\:{\mathbf{P}}_{j}$:

2 $$\:{k}_{j}=\mathcal{K}\left(\widehat{\mathbf{W}}\left({\mathbf{c}}_{j}\right),\widehat{\mathbf{W}}\left({\mathbf{c}}_{j+1}\right),{\mathbf{P}}_{j},{\upalpha\:}\right)$$

**Fig. 8:**
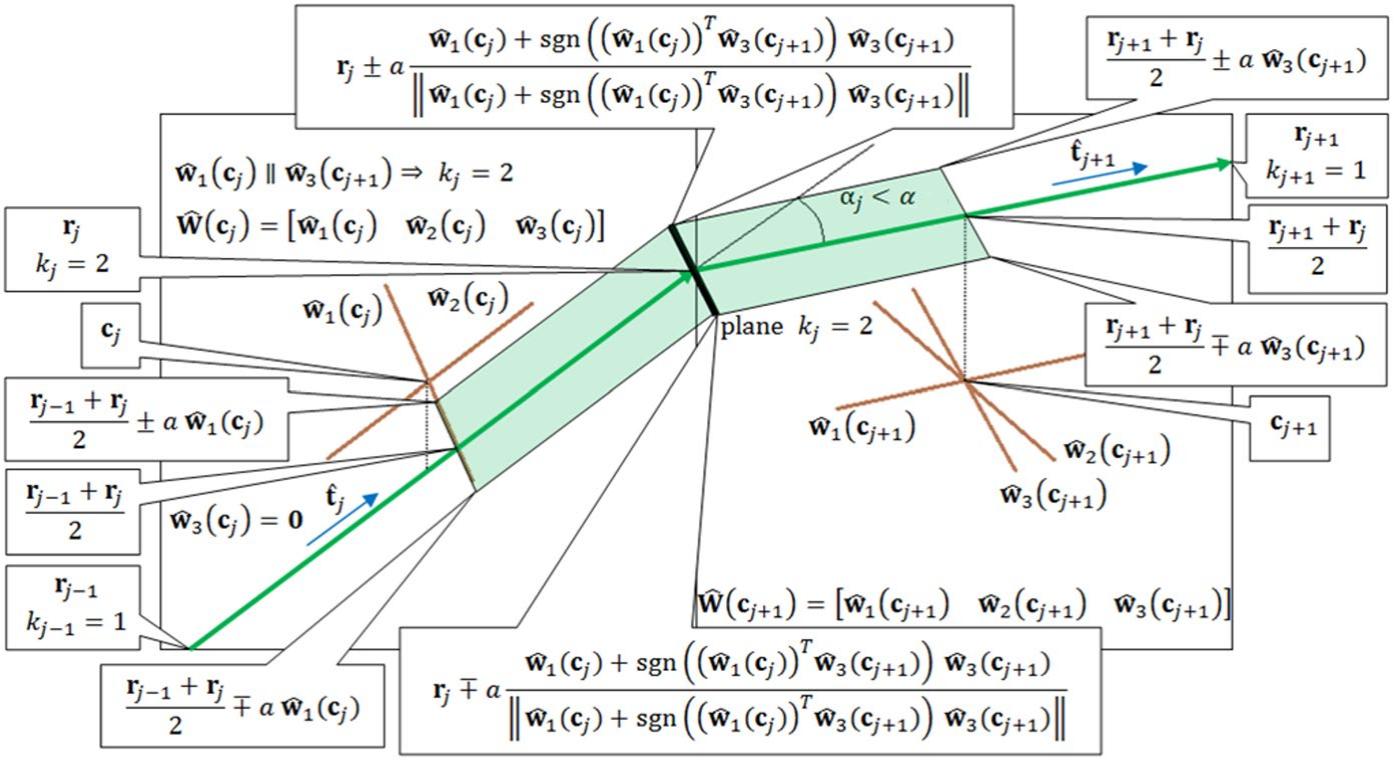
Full representation of a plane *k*_*j*_=2 as a piecewise plane. The piecewise plane is divided by 2 semi-planes: the first semi-plane is parallel to the direction **t̂**_*j*_ and the second plane is parallel to the direction **t̂**_*j*+1_. The coordinates of vertex of planes and the intersection between the propagation line and the edges of semi-planes are identified by balloons. For *k*_*j+1*_=3, it is represented by 3 planes, 2 planes parallel to propagation line and often almost orthogonal between themselves, and 1 plane often almost orthogonal to the propagation line. The green line is the propagation line, the blue arrow is the direction of **t̂** which defines the propagation direction of the green line, and the brown lines are the directions of axes systems with one of those axis directions generating the **t̂**.

If *k*_*j*_=2, one piecewise plane is drawn along the piecewise line going from (**r**_*j*–1_+ **r**_*j*_)/2 to (**r**_*j*+1_+ **r**_*j*_)/2. This piecewise plane is divided into two planes; one is in voxel **c**_*j*_, it is parallel to the direction of **t̂**_*j*_ and going from (**r**_*j*–1_ + **r**_*j*_)/2 to **r**_*j*_; and the other plane is in voxel **c**_*j*+1_, it is parallel to the direction of **t̂**_*j*+1_ and going from **r**_*j*_ to (**r**_*j*+1_+ **r**_*j*_)/2. If *k*_*j*_ = 3, then two piecewise planes are drawn along the piecewise line going from (**r**_*j*–1_ + **r**_*j*_)/2 to (**r**_*j*+1_+ **r**_*j*_)/2, and three planes typically almost-ortogonal to the propagation piecewise line and almost parallel between themselves are also drawn, with these three planes intersecting the propagation piecewise line in points (**r**_*j*–1_ + **r**_*j*_)/2, **r**_*j*_ and (**r**_*j*+1_+ **r**_*j*_)/2, respectively, in a total of 7 planes (4 parallel, and 3 non-parallel to the propagation direction). To draw a plane, a closed polygon is drawn, by crossing a sequence of points. The face of this polygon is colored using the absolute average color of the four polygon edge directions, divided by the infinity-order norm of itself. For the planes non-parallel to the propagation direction, the polygon constraining the plane is a parallelogram and the four edge directions are two pairs of axes systems. For the planes parallel to the propagation direction, the polygon constraining the plane is a scalene trapezoid and the four edge directions are a pair of axes systems plus 2 line segments parallel to the propagation direction. For each pair of axes systems directions, one element of the pair is associated to voxel **c**_*j*_ and the other to voxel **c**_*j+1*_ (see Fig. 8).

## Data Availability

The datasets used and analyzed during the current study are available from the corresponding author on reasonable request. Datasets used were provided by the Human Connectome Project, MGH-UCLA Consortium (Principal Investigators: Bruce R. Rosen, Arthur W. Toga) funded by the National Institutes of Health, NIH Blueprint Initiative for Neuroscience Research grant U01MH093765 and National Institutes of Health grant P41EB015896. Datasets analyses were made using in-house Matlab software.

## Code availability

The Matlab software and Dataset together with the corresponding Gridography Instruction Manual can be found at the DOI: 10.5281/zenodo.17714059.
